# Evolution of public funding since primary care research was considered as a priority research domain in france

**DOI:** 10.1186/s12875-024-02384-7

**Published:** 2024-04-27

**Authors:** Jordan Scheer, Taylor Leroy, Sylvie Guillo, Florence Tubach, Antoine Rozès, Amandine Verga-Gérard, Francis Guillemin, Maryse Lapeyre-Mestre, Anthony Chapron

**Affiliations:** 1 RECaP F-CRIN Inserm network (The RECaP/F-CRIN network (Réseau de Recherche en épidémiologie clinique et en santé publique/French Clinical Research Infrastructure Network) is a French research network supported by Inserm and F-CRIN (French Clinical Research Infrastructure Network), https://recap-inserm.fr/home.html?, Allée du Morvan, Vandoeuvre-lès- Nancy, F-54500 France; 2 Department of General Practice, Rennes University, Centre Hospitalier Universitaire (CHU) de Rennes, Rennes, France; 3 Centre Hospitalier Universitaire (CHU) de Rennes, Rennes University, Institut National de la Santé et de la Recherche Médicale (INSERM), Rennes, CIC-1414 France; 4 CHRU, Inserm, Université de Lorraine, CIC Epidémiologie clinique, Nancy, France; 5 Ecole des hautes études en santé publique (EHESP), Rennes University, CHU Rennes, INSERM, Institut de recherche en santé, environnement et travail (IRSET) - UMR_S 1085, Rennes, France; 6 Département de Santé Publique, Centre de Pharmacoépidémiologie (Cephepi), Sorbonne Université, INSERM, Institut Pierre Louis d’Épidémiologie et de Santé Publique, AP-HP, Hôpital Pitié Salpêtrière, Paris, CIC-1901 France; 7 Service de Pharmacologie Médicale et Clinique, Faculté de Médecine, CHU, Université de Toulouse, CIC 1436, Toulouse, France

**Keywords:** Primary care, Clinical research, General medicine, Research methodology, Public funding

## Abstract

**Purpose:**

Annually, the French Ministry of Health funds clinical research projects based on a national call for projects. Since 2013, the Ministry has prioritized funding of primary care. Projects selected for funding are made public without distinguishing the specific area of research. The objective of this study was to identify and describe the evolution of the primary care research projects funded by the Ministry of Health between 2013 and 2019.

**Method:**

We reviewed all of the 1796 medical research projects funded between 2013 and 2019 and categorized projects as primary care projects by using a list of specific keywords. This list was established through two approaches: (1) selected by an expert committee, the RECaP primary care working group, and (2) using an automated textual analysis of published articles in the field. The keywords were used to screen the titles of the medical research projects funded. The abstracts (at www.clinicaltrials.gov) or details (from project leaders) were then analyzed by two independent reviewers to determine true primary care projects.

**Results:**

Finally, 49 primary care projects were identified, representing 2.7% of all medical research projects funded, without any significant change over the period. These projects were predominantly interventional (69%), with a median number of patients expected per project of 902.

**Conclusion:**

Despite the prioritization of primary care research in 2013 by the French ministry of health, the number and proportion of projects funded remains low, with no significant change over the years.

**Trial registration:**

Not applicable.

**Supplementary Information:**

The online version contains supplementary material available at 10.1186/s12875-024-02384-7.

## Background

In the last 2 decades, research in primary care has been considered as a cornerstone for acquiring scientific knowledge in this field [1, 2] and for improving healthcare worldwide, and specifically in France [3, 4]. The importance of a care system based on primary care, for example, is helping to reduce mortality in the population [5]. Two French national reports, in 2006 [6] and 2015 [7] highlighted the pivotal role of general medicine and primary care research to reinforce French healthcare system. Primary care is everyone’s local entry point to healthcare system ensuring accessible, integrated, and continuous care. Moreover, primary care professionals (general practitioners as well as nurses, physiotherapists, etc.) often coordinate services critical for more specialized care [8]. The development of an academic research in primary care is a milestone for reinforcing the role of general medicine. This highlights the pivotal role of primary care, not only as the entry point, but also throughout patient healthcare.

In France, since 1992, the French ministry of health - via a directorate called the “Direction Générale de l’Offre de Soins (DGOS)” - launches a call for proposals annually, with the aim to promote the French clinical research based on a national call for projects named Clinical Research Program. This call for proposals was initially dedicated to clinical research carried out in French public university hospitals, and then gradually expanded to support projects contributing to medical progress as well as to the improvement of the quality of care, the efficiency of the healthcare system, and the evaluation of a medical or organizational innovation. Since 2013, the DGOS prioritized primary care research projects for grants from the Ministry. To ensure confidentiality and to offer the opportunity of a future submission the number of projects considered as targeting the field of primary care submitted or funded and the details concerning these projects are not publicly disclosed (as well as for projects in other fields). They only disclose the titles and the leaders’ names of all medical research projects funded.

We hypothesize that the prioritization of primary care research beginning in 2013 has promoted the emergence of primary care projects, with a progressive increase of funded projects in this field over time. However, the public evaluation of the impact of the national funding policy that since 2013 has prioritized primary care research projects has not yet been performed.

The objective of the study was to identify and describe the evolution of primary care research projects funded by the clinical research programme supported by the French Ministry of Health, between 2013 and 2019.

## Methods

### Setting

The Research in Clinical Epidemiology and Public Health (RECaP) French network implemented a specific working group, comprised of 11 researchers from diverse fields and backgrounds working in primary care research, to focus on this field.

In 2018, the RECaP primary care working group proposed an operational definition of primary care research in order to promote the research in this field in France [9]. This definition taking into account professional and practice settings allowed the field of research in primary care to extend beyond general practice.

For this, the RECaP group listed the common core criteria that define primary care [10, 11]. This list specified four dimensions that define primary care: first contact, continuity, coordination, and comprehensiveness of care and provided details concerning the health professionals involved and specific settings (medical practices, health centers, community pharmacies, etc.). The list is shown in Table S2 in the supplementary appendix.

### Study design

An observational study based on public information available on the French Ministry of Health website, related to research and innovation in health was conducted. We reviewed the 1796 medical research projects funded by the DGOS between 2013 and 2019. We started the analysis in 2013, because prior to this, the database is not exhaustive and consequently not reliable for identifying projects. We decided to limit the analysis to projects prior to 2019 due to the occurrence of the coronavirus disease 2019 (COVID-19) that disrupted the call for projects for 2020.

We have deliberately excluded calls for projects concerning oncology and translational research since we considered these outside the scope of primary care. We verified our assumption by randomly assessing 10% of these projects out of the database.

### Data sources

We used data that were publicly available from the French ministry of health website (https://sante.gouv.fr/systeme-de-sante/innovation-et-recherche/l-innovation-et-la-recherche-clinique/appels-a-projets/article/les-projets-retenus#PHRC). Each year, the lists of funded projects are provided (in French), with only the title of the project, its acronym, the name of the project leader, the institution in charge of funding management, and the total funding. In France, only funded studies are made public, but not studies that have been submitted to calls for project funding. Similarly, the categorisation of research projects by discipline is not made public.

To have more information about the funded projects we decided to search the abstract of these projects at *ClinicalTrials website* using the acronym of the project and/or the investigator name. For projects not recorded/registered on this registry, we contacted the corresponding project leaders to obtain details on the study (there were up to 2 solicitations per email with a reminder at 3 weeks if there was no initial response). The following data were requested from the project leaders: study description, study design, condition or disease, intervention, criteria for judgement, inclusion and exclusion criteria.

Information concerning the projects submitted, but not granted funding, was not publicly available (to ensure confidentiality and to offer the possibility of a future application for funding).

### Definition of primary care projects

Projects were considered as primary care if they had at least one keyword related to primary care in their title and their abstract met the criteria of the RECaP primary care working group [9].

### Identification of the discriminant primary care keywords

Keywords associated with primary care were identified using two complementary approaches.

The first approach was based on keywords derived from the characteristics of the field of primary care as proposed by the RECaP primary care working group [9].

In addition, a second list of keywords compiled of the most frequent words used in the titles of the original articles in English language published between 2013 and 2019 in either the 19 primary care journals recorded in the Journal Citation Reports in 2021 and in the French journal of general medicine “*Exercer*”, by use of a N-gram model [12] to automate word selection using R© software. The purpose of this second approach was to identify and select the most frequent word groups used in titles of articles published in the primary care research field in France and internationally. The process used to define keywords is shown in Figure S1 in the supplementary appendix.

### Screening and Inclusion

Initially, titles of projects were manually screened and projects whose title included at least one of the above-mentioned keywords were selected. Titles of all the funded projects were read without going through automation based on the identified keywords.

Second, all the abstracts of projects selected in this first step were screened using the list of the RECaP primary care working group [9]. Projects that addressed research questions meeting the RECAP’s list were definitively considered as being primary care. If the abstract met at least one of the 4 dimensions of the list: the project was considered primary care.

Projects were classified as primary care and included independently by two RECaP working group members (JS, TL). In cases of discordance, a third member (AC) was solicited to resolve discrepancies.

Among the projects initially excluded based on their titles, 5% were randomly selected. The abstracts of these selected projects were verified, using the identified keywords, to ensure that they were not primary care projects.

Projects for which the abstracts were not registered at www.clinicaltrials.gov or not provided by the project leader (despite 2 requests for data by email) were excluded from the analysis.

### Description of primary care research projects

For the primary care projects identified, data were extracted independently, using a standardized report form, from the DGOS website and the *ClinicalTrials.gov registry*. The following project characteristics were extracted using a standardized form: project title, name and specialty of the project leader, year of financing, primary objective, design, number of patients recruited, characteristics of eligible patient (age, sex, condition), methods for recruiting patients, and if projects were indexed as primary care in the *ClinicalTrials.gov registry*.

A member of the RECaP working group (JS) categorized each selected project according to the 17 body system chapters of the second edition of the International Classification of Primary Care (ICPC-2) [13].

### Statistics analysis

The data were described as numbers and percentages. The evolution of number of primary healthcare projects funded between 2013 and 2019 was described performed using a Jonckheere trend test. Analyses were performed using SAS©.

## Results

### Identification of keywords for primary care

Overall, a list of 31 keywords was defined to identify projects in primary care from the 1796 medical research projects granted funding by the DGOS between 2013 and 2019. Among these, 23 resulted from the proposal of the RECaP working group, and a further 8 from the automated textual analysis. The final list of retained keywords is shown in Table S1 in the supplementary appendix.

To verify that projects excluded based on their titles using the identified keywords were not primary care projects, we randomly selected 79 projects (5% of those initially excluded). Analysis of the abstract of these projects confirmed that none of them concerned primary care.

### Screening and inclusion of primary care research projects

**Fig. 1:**
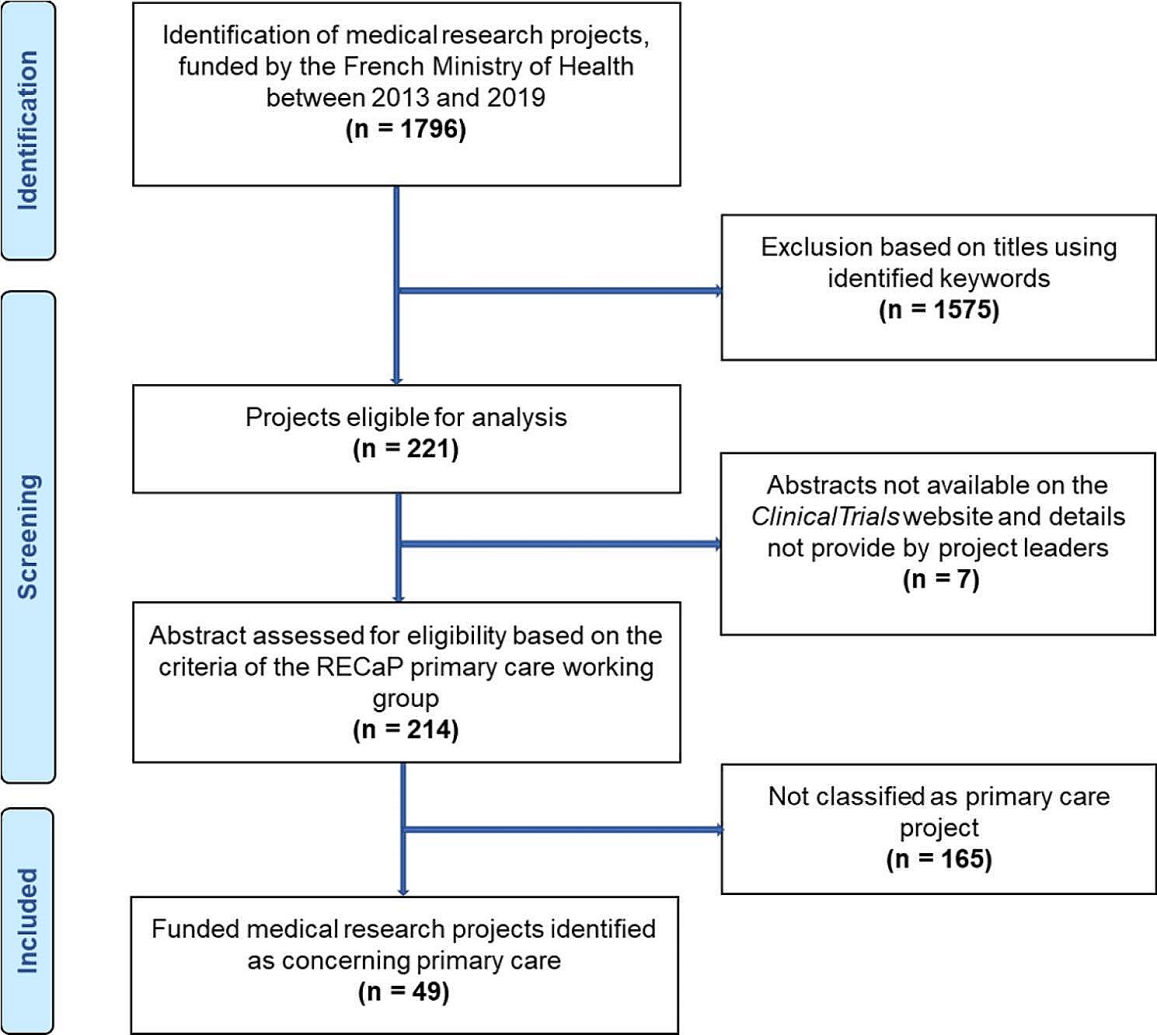
Flow chart for identifying primary care projects funded by the French Health Ministry between 2013 and 2019

Among the 1796 medical research projects granted between 2013 and 2019, 49 (3%) were considered as primary care projects and were included in our study. The list of the 49 projects included is shown in Table S3 in the supplementary appendix. The screening process is presented in Fig. 1.

### Evolution of funded primary care projects

**Fig. 2:**
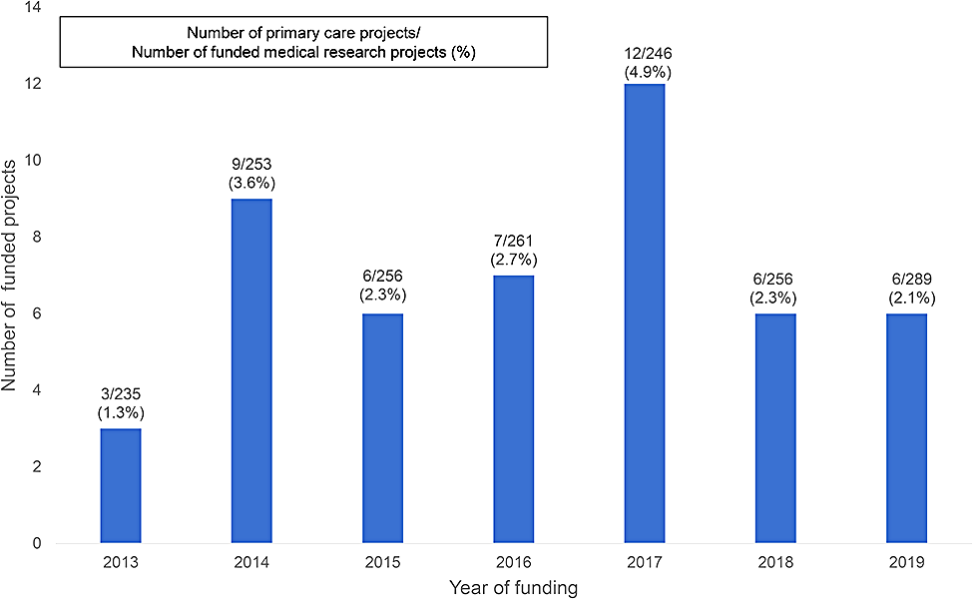
Numbers of primary care projects financed by French Health Ministry between 2013 and 2019

Between 2013 and 2019, the highest annual number of projects funded was 12 projects (5%) in 2017. After 2017, fewer projects were funded: 6 (2%) in 2018 and 2019. The annual numbers of projects funded by the DGOS are presented in Fig. 2.

The evolution of the annual number of projects funded between 2013 and 2019 was not statistically significant (*p* = 0.7). Concerning the design of the primary care projects, 34 (69%) were interventional and 15 (31%) were observational.

### Projects’ characteristics

Among the 15 observational studies, 14 (93%) were cohorts (of which 11 were prospective cohorts) and one (7%) cross-sectional. The median budget allocated per project was 405 800 € (Q1: 232 160; Q3: 627 600).

Overall, 28 (57%) were coordinated by doctors practicing other specialties (not general practitioners), 13 projects (27%) by general practitioners, 4 (8%) by pharmacists, 2 (4%) by nurses, 1 (2%) by a dentist, and 1 (2%) by an occupational therapist.

Most projects, 9 (18%), were coordinated by a project leader based in Paris. Among the 32 medical universities in France, 14 had at least one primary care project funded by the DGOS during the period studied.

### Patients’ characteristics

**Fig. 3:**
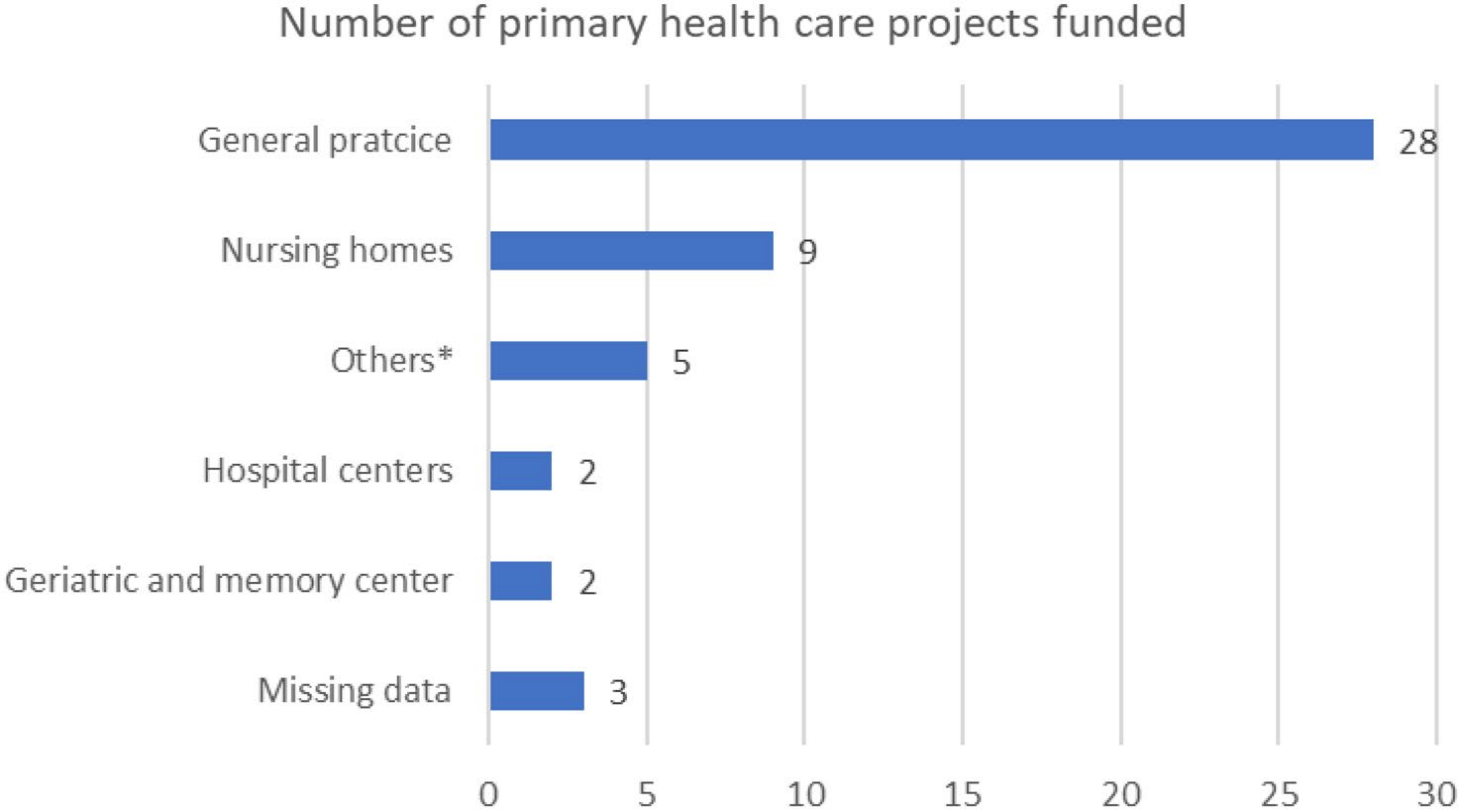
Location of patient recruitments for the primary care projects funded between 2013 and 2019 by the french ministry of health. *Other locations of recruitment were by mail (*n* = 1), at a mental healthcare establishment (*n* = 1), using data from the National Health Insurance System database (“Système National des Données de Santé”, SNDS) (*n* = 1), at the housing center coordinated by the city council (*n* = 1), and at pediatric consultations (*n* = 1)

Among the 49 selected projects, the median number of patients expected per project was 902(Q1: 300; Q3: 2193). Four projects (8%) only woman, 11 (22%) only older people, 4 (8%) only children, 1 (2%) recruited patients older than 15 years, 21 (43%) patients 18 years or older, and for 12 (24%) the age limits were not specified. Planned recruitments were predominantly in general practice offices for 28 projects (57%), followed by nursing homes for 9 projects (18%), see Fig. 3. One project (2%) was based on the National Health Insurance System database (Système National des Données de Santé, SNDS), a national electronic healthcare database, including all health care reimbursements in ambulatory and hospital settings and causes of death for 67 million of people living in France [14, 15].

### Scope of the projects

The primary objectives of the projects were prevention for 16 projects (33%), treatment evaluation for 11 projects (23%) diagnostic and health services research for 10 projects respectively (20%) and screening for 2 projects (4%).

**Fig. 4:**
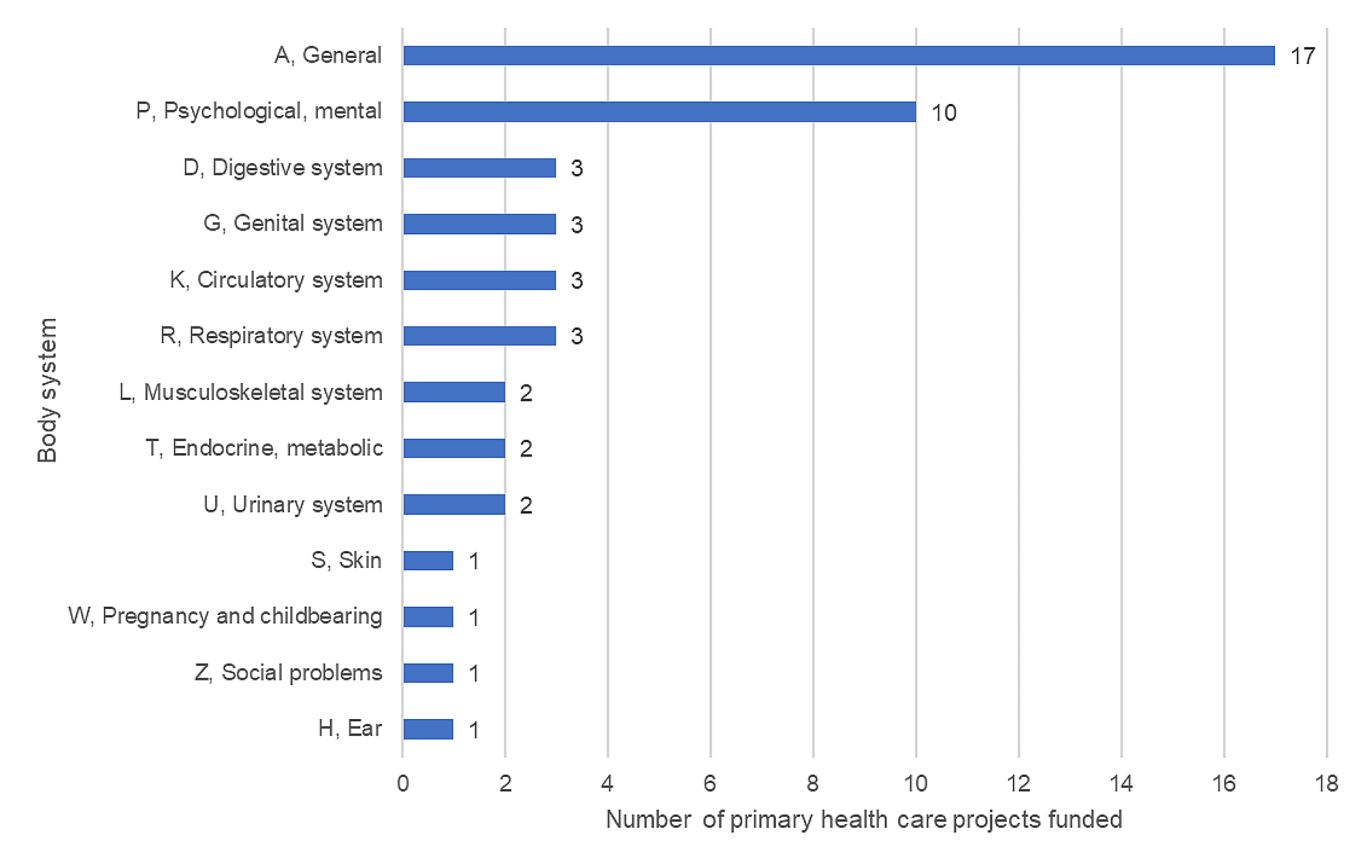
Classification of the funded primary care research projects into body systems according to ICPC-2 [13]

According to the ICPC-2 classification [13], by body systems, 17 projects (35%) were classified as general conditions and 10 (20%) as psychological and mental conditions. The classifications of all projects by ICPC-2 are presented in Fig. 4.

### Indexing on the *ClinicalTrials* website and publication

Twelve projects (24%) were indexed as primary care on the *ClinicalTrials* website. In March 2022, among the 49 projects, 9 (18%) had a publication(s) indexed on the *ClinicalTrials* website.

## Discussion

The prioritization of primary care research is an important step towards promoting research in this field. Despite prioritization by the DGOS since 2013, only 3% of the projects funded publically between 2013 and 2019 were classified, by our research, as primary care projects.

The annual proportion of primary care projects remains small and has not changed over time. Unfortunately, the number of projects that requested primary care funding was unknown. We are thus unable to determine whether the small proportion of primary care projects funded reflects the limited number of projects that requested funding or whether numerous primary care projects were rejected by the scientific committee evaluating the projects submitted. In fact, the government has not made available summary statistics for funded projects classified as primary care. Summary data concerning the number of projects identified as primary care, the number of primary care projects funded per year and the percentage of funding also were not made available. Consequently, it would to be of interest, for transparency, that the description of the projects that requested funding be made publicly available.

Moreover, since the data on the DGOS website was not exhaustive before 2013, it was not possible to compare the number of primary care projects funded before and after 2013, the date on which primary care was first prioritized for funding.

Our study concerned projects funded by the French Ministry of Health at the national level. However, we did not include projects funded at the regional level since the list of these projects was not publically available. We recommend that details concerning regionally funded projects be publically available, to promote greater transparency.

The main strength of our study is to provide original data and a standardized method to assess funded primary care projects, in this context, of lack of public data and transparency. Another strength is the innovative method developed to identify primary care projects using a dedicated compiled list of keywords (*Figure **S1* in the supplementary appendix). This dedicated list, derived using two complementary approaches (keywords from an expert group and from an automated textual analysis) allows for an exhaustive and reproducible selection of the projects based on their titles. The initial selection of primary care projects was performed by verifying the presence of these keywords in the project titles. It is possible that certain primary care projects were not selected because these keywords were not present in their titles. Among the 221 projects initially selected based on these keywords, only 49 projects were finally classified as primary care projects.

Another strength is our analysis of clinical trials. It is noteworthy that 8 projects identified in our study were not indexed, by the project leader, as primary care on the *ClinicalTrials* website. This may hinder the identification of primary care projects. We would welcome a more comprehensive indexing of these projects on the *ClinicalTrials* website to facilitate their identification.

Various factors that concern the organization of the French healthcare system may explain why so few primary care projects are funded despite the prioritization of their funding. Only recently, has general medicine been integrated as a discipline in French universities. Outside of the university system, general practitioners have little time or encouragement to do research. Furthermore, digital medical files differ among general practitioners making it difficult to collect standardized data. At present, the network of investigators doing research in primary care is limited.

When the scientific committee assesses the projects submitted, the thematic prioritization of projects allows primary care projects of equivalent scientific quality to be favored. Our results suggest that a dedicated financing of primary care projects may be more effective for promoting this area of research.

Internationally, other countries have introduced different methods to prioritize funding of research in primary care [6]. In the United Kingdom, following a report published by Prof. David Mant in 1997 entitled, “R&D in primary care”, 5 million pounds were invested in the training healthcare professionals in research and for creating research infrastructures. Moreover, 12 million pounds were allocated to fund research projects. Also, a national primary care research centre was created. In the Netherlands, there is a programme that funds doctorates for general practitioners.

Despite these different initiatives to fund and promote primary research, relatively few primary care publications, among medical research publications, are published. In 2019, a study reported that primary care publications indexed in Medline represented only a minor proportion of all publications: 9% of publications in the United Kingdom, 6% in Canada, 6% in Australia, and 5% in the United States [16]. This was already highlighted in an article published in 2005: indeed, for every article published in primary care, 20 were published in cardiology [17]. However, the number of articles from French research in primary care continues to increase in English-language literature [18], despite a low rate of research projects funded by the Ministry. In particular, these works stem from academic theses in general medicine generally conducted without specific funding.

Primary care research seems to be more developed in countries with more structured primary care systems. Indeed, the extent of primary care research depends on the importance of primary care in the healthcare system and in medical universities, and to the existence of a structured, national system to collect and store primary care data [19]. Also, primary care research is more developed in countries where healthcare professionals are more implicated in and informed about research.

In our review, we note that not all patients are included in primary care, but that the study is conducted in primary care settings and includes primary care professionals or collaborations between primary care and secondary sector of cares. The diversity of recruitment methods and fields observed in the projects shows that primary care research is characterized by coordination between diverse healthcare professions in different fields, which reflects the richness of primary care research. This diversity of coordination or partnerships has already been reported in various studies [1].

It is important to continue to promote primary care projects through prioritized funding.

However, we do suggest a more transparent project selection process to allow an evaluation of the number of projects submitted and selected and ensure that the project declared as being primary care are within the scope of primary care. In 2021, the French Ministry of Health did introduce a new call for proposal dedicated to primary care at the regional level. This may be a more effective solution for prioritizing and funding primary care research. The effectiveness of this new strategy still needs to be evaluated.

## Conclusion

Despite the prioritize funding of primary care projects, in France since 2013, only relatively few of the medical research projects funded correspond to primary care projects. A more transparent selection process and alternative methods of promoting primary care research are still needed.

### Electronic supplementary material

Below is the link to the electronic supplementary material.

Supplementary Material 1

## Data Availability

The full data set is available for review upon reasonable request from the corresponding author.

## Abbreviations

ICPC-2: the second edition of the International Classification of Primary Care
COVID-19: coronavirus disease 2019
DGOS: “Direction Générale de l’Offre de Soins”: the directorate under the responsibility of the French Ministry of Health concerned with the provision of healthcare
RECaP: Research in Clinical Epidemiology and Public Health
SNIIRAM, SNDS: “Système National des Données de Santé”: the French National Health Insurance System database
