# Supplementary material for: Evolution of public funding since primary care research was considered as a priority research domain in france
Source: BMC Prim Care. 2024 Apr 27;25:142. doi: 10.1186/s12875-024-02384-7 (PMC11055352; doi:10.1186/s12875-024-02384-7)
Supplement: Supplementary file 1 — Supplementary Material 1 [file 12875_2024_2384_MOESM1_ESM.docx]

**SUPPLEMENTARY APPENDIX**


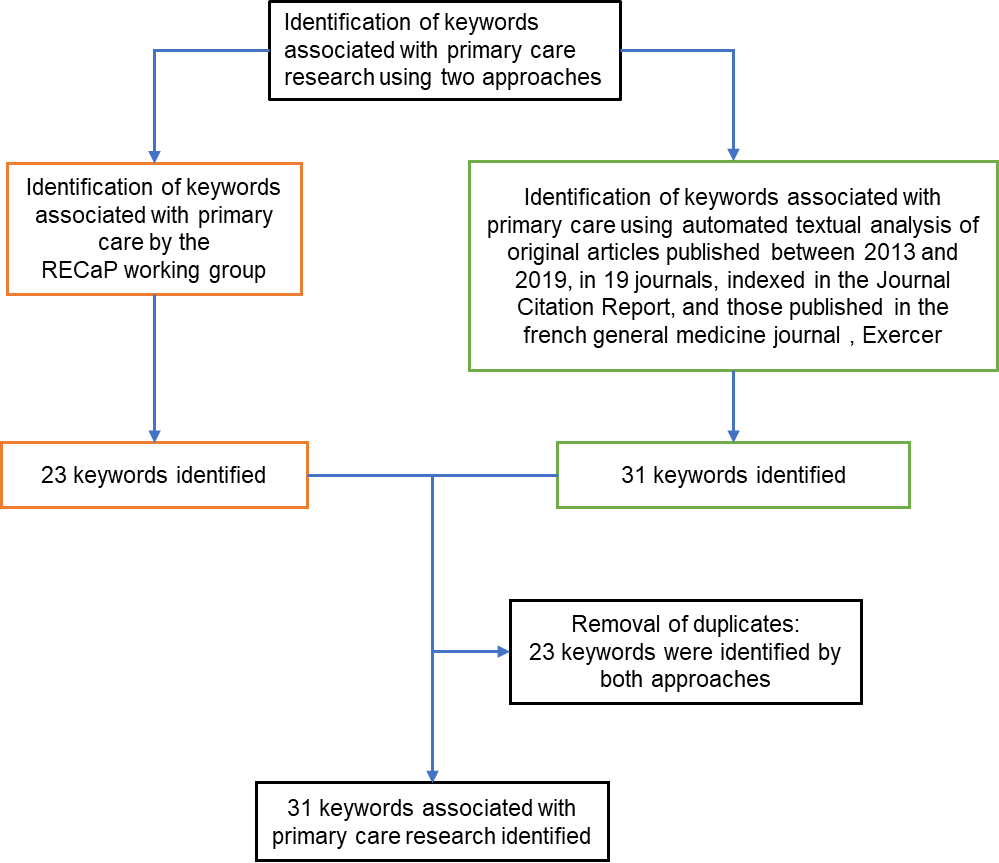


**Figure S1. Process for identifying keywords associated with primary care research using two complementary approaches.**

**Table S1. Keywords associated with primary care research.**

| **23 Keywords associated with primary care research identified by the RECaP working group and by the automated textual analysis** | **8 Keywords only identified by the automated textual analysis** |
| --- | --- |
| Primary Care  Primary healthcare  General Practitioner  City  Pharmacist  House  Ambulatory  Nurse  Dentist  Physical Therapist  Midwife  Pediatric nurse  Maternal and child welfare  First resort  Office  Prevention  Home  Care pathway  Multidisciplinary  Retirement homes/care homes/nursing homes  Telemedicine  Community  Prison | Family  Physician  Internship  Diagnostic  Patient-centered  Population  Prescription  Practice |

**Table S2. Table of definition of primary care according to the associated healthcare profession and where they practice.**

| **Characteristic** | **Definition** | **Profession associated (Who?)** | **Location associated (Where?)** |
| --- | --- | --- | --- |
| First contact | Physical, geographical, temporal, and financial accessibility.  Welcomed without any discrimination, irrespective of the age, living circumstances, and population. | Healthcare professional (prescriber): general practitioners, dentists, midwives, specialists that are directly accessible (psychiatrists, pediatricians, gynecologists, and ophthalmologists).  Paramedical professionals: certified nurses, massage therapists-physiotherapists, and pediatric nurses. | Private practice  Multidisciplinary medical clinics  Medical centers  Pharmacies  Emergency services  Permanent medical services  University health services  Prison health services  SOS medicine (French emergency service at patient’s home)  Humanitarian and associations (“Medécin du monde”)  Screening centers (anonymous and free)  Family planning centers  Biological testing laboratories  Mother and child protection centers |
| Continuity of healthcare | Guarantee continual healthcare over time.  Developing a lasting relationship, based on confidence, between caregiver and patient over time, a major criterion for the quality of care. | General practitioners  Certified nurses  Pharmacists  And depending on the healthcare organization of the region: dentists; midwives, pediatricians, psychiatrists, gynecologists, and ophthalmologists. | Private practice  Multidisciplinary medical clinics  Medical centers  Prison health services  Mother and child protection centers |
| Coordination | Situation at the beginning of the healthcare pathway, coordination of the various role plays, intersection between healthcare for individual patients and public healthcare programs. | The healthcare professional declared as the patient’s reference doctor (initially general practitioner or pediatrician). | The place of practice of the patient’s reference doctor. |
| Global healthcare | Global healthcare of the patient, from biological, psychological, and social perspectives.  Not focusing on a specific aspect of healthcare, in contrast to secondary and tertiary healthcare.  Considering the community dimension. Proposing of a variety of first response services that responds to most of the healthcare needs of the population. | General practitioners  Certified nurses  Pediatricians | Private practice  Multidisciplinary medical centers  Medical centers |

**Table S3. Table of the 49 primary care projects funded by the French Ministry of Health between 2013 and 2019.**

| **English project title** | **Year of funding** |
| --- | --- |
| Benefit/Risk in Real Life of New Oral Anticoagulants and Vitamin K Antagonists in Patients Aged 80 Years and Over | 2013 |
| Impact of On-site Evaluation of Substances Consumption on Opiate Maintenance in the Context of Family Practice | 2013 |
| Assessment of a Multifaceted Risk Management Program in French Multiprofessional Offices in Primary Care | 2013 |
| MOther-Child Interaction Assessment TRAINING for Pediatricians | 2014 |
| Medico-economic Evaluation of Complicated Obesity in Primary Care | 2014 |
| Participation in Screening for Cervical Cancer: Interest of a Self-sampling Device Provided by the General Practitioner | 2014 |
| Compared Efficacy of Nurse-led and GP-led Geriatric Assessment in PrImary Care | 2014 |
| Patient Care Pathways Between Ambulatory and Hospital Settings in Mental Health Care | 2014 |
| Impact of Interprofessional Training and Co-ordination on Early Identification and Proactive Approach to End-of-life Situations in the Context of Primary Care | 2014 |
| Advanced Nurses vs. General Practitioners for the Management of Outpatient Alcohol Detox: a Safety and Cost Comparison | 2014 |
| Factors Predisposing to Inappropriate Transfers of Nursing Home Residents to Emergency Departments (FINE) | 2014 |
| Mortality and Economic Impact of Stopping Statins in People Aged of 75 and Over: a Pragmatic Clinical Trial | 2014 |
| Elderly Patient at Risk of Loss of Mobility, Exercise - Primary Care, Prevention, Care Pathways | 2015 |
| A Quality Rating Scale for Patients in Complex Situations and Their Caregivers | 2015 |
| Efficacy of Information System Regarding the Consumption of Antibiotics and Bacterial Resistance in Primary Care | 2015 |
| Optimizing Access to Care Through New Technologies: a Randomized Study Evaluating the Impact of Telephone Contact and the Sending by the General Practitioner of Suspicious Lesions Melanoma Photographs | 2015 |
| Prevention of Diseases Induced by Chlamydia Trachomatis | 2015 |
| Potentially Avoidable Hospitalisation in France | 2015 |
| Primary Dependence to Analgesic Drugs | 2016 |
| Impact of the Reduction in Antihypertensive Treatment on Total Mortality in Frail Subjects With Low Systolic Blood Pressure: Study in Subjects Over 80 Years Living in Nursing Homes | 2016 |
| Cost-utility Analysis, Cost-effectiveness Analysis, Budget Impact Analysis | 2016 |
| Evaluating the Effectiveness of a New Way of Organizing Primary Health Care to Improve the Management of Alzheimer's Disease | 2016 |
| Effectiveness and Organizational Conditions of Effectiveness of Telemedicine in Establishments Providing Care for the Dependent Elderly | 2016 |
| Validation of a Specific Tool Scoring Residents at Risk of Escaping/Elopement From Nursing Homes | 2016 |
| Efficacy of the Buzzy® Device on the Prevention of Health Care Induced Pediatric Pain in a Vaccination Center | 2016 |
| Adolescent Depression Associated With Parental Depression | 2017 |
| A Collaborative Approach to Medication Reviews for Older Patients With Polypharmacy | 2017 |
| Impact of a Communication Toolkit on Antibiotic Prescribed by General Practitioners: a Randomised Trial | 2017 |
| Impact of the Use of CRP on the Prescription of Antibiotics in General Practitioners` | 2017 |
| Blood Fibrocytes During an Exacerbation and Lung Function Decline in Patients With COPD in Primary Care | 2017 |
| Identification of neurocognitive disorders by general practitioners in primary care | 2017 |
| Prevention of the Older Adult's Loss of Autonomy at Home Through a Targeted Physical Exercise Program | 2017 |
| Use of a Share Decision Making Tool in the Care of Acute Cystitis Without Risk of Complication in Primary Care | 2017 |
| Medico-economical Evaluation on Buccodental Teleexpertise in Nursing Home | 2017 |
| Risk Factors for Falls After Intervention of the Urgent Medical Assistance Service (SAMU) in the Elderly Person at Home | 2017 |
| Improving Access to Psychiatric Care for Patients in Primary Care | 2017 |
| Prevention of pneumonia in Nursing Home | 2017 |
| Effectiveness of an Innovative Program in Primary Care for Vulnerable and Precarious Population With Access Barriers to Healthcare System: PASS de Ville | 2018 |
| Impact on Antibiotic Prescriptions of a Bundle Intervention Conducted by Medical Representatives in General Practitioner Facilities, Based on Operational Demonstration of an Internet Decision Support | 2018 |
| General Practitioners and Participation Rate in ColoRectal Cancer Screening | 2018 |
| Combined Incentive Actions, Focusing on Primary Care, to Improve Cervical Cancer Screening in Women Residing in Socio-economically Disadvantaged and Untracked Geographical Areas: a Hybrid Efficacy and Implementation Trial | 2018 |
| Sustainability of a Research Program in Risk Management | 2018 |
| Screening in Primary Care of Advanced Liver Fibrosis in NAFLD and/​or Alcoholic Patients (SOPRANO) | 2018 |
| Combined Incentive Actions, Focusing on Primary Care, to Improve Cervical Cancer Screening in Women Residing in Socio-economically Disadvantaged and Untracked Geographical Areas: a Hybrid Efficacy and Implementation Trial | 2019 |
| Efficacy of a Multi-faceted Intervention to Deprescribe Proton Pump Inhibitors (PPI) in Primary Care: a Population-based, Pragmatic, Cluster-randomized Controlled Trial | 2019 |
| International Normalised Ratio Evaluation by Generalist Practitioners in Full-time Care Establishments for the Elderly | 2019 |
| Interventional Research With Mixed Methods on an Early Integrated Palliative Approach in Nursing Home | 2019 |
| Efficiency of E-learning and Role-playing for the Training of Nursing Home Caregivers in the Support of Agitation in Neurodegenerative Diseases | 2019 |
| Impact on Antibiotic Prescriptions of a Bundle Intervention Conducted by Medical Representatives in General Practitioner Facilities, Based on Operational Demonstration of an Internet Decision Support Tool: Antibioclic | 2019 |
